# Sagittal and axial mobility of 1st ray in hallux valgus

**DOI:** 10.1186/1757-1146-7-S1-A133

**Published:** 2014-04-08

**Authors:** Kiwon Young, Jin Su Kim, Hun ki Cho, Hyoung Suk Kim

**Affiliations:** 1 Dept. of Orthopaedic Foot & Ankle Eulji Medical College Hospital Nowon, Seoul, Korea

## Introduction

Hypermobility of the first ray is one causative factor in development and recurrence of Hallux valgus[1],[2],[3]. so treated as an important factor in hallux valgus. While most discussions of 1st ray instability refer to sagittal motion [4],[5], [6],[7]. Increased motion may also be present in the axial plan. However, there is no known way of measuring motion of axial plan. In this study, we assessed the axial plan mobility by means of measuring the difference between weight bearing IMA and non-weight bearing IMA from foot AP radiograph. This study investigated the difference between the axial motion of the first ray of the symptomatic hallux valgus patients group and that of the normal group.

## Methods

A group of 108 women with symptomatic hallux valgus and 37 control women, age 21 to 84 years were measured weight bearing and non-weight bearing IMA and calculated the difference. We measured the 1st ray sagittal range of motion by the EMC device [4]. We moved the first ray up and down and recorded the distance (d). We also measured the first metatarsal length (l) on the AP foot x-ray film. Finally, we calculated the 1st ray range of motion (A) using the above data. There data was statistically annalize with correlation with HV angle, IM angle and each other.

## Result

**Figure 1:**
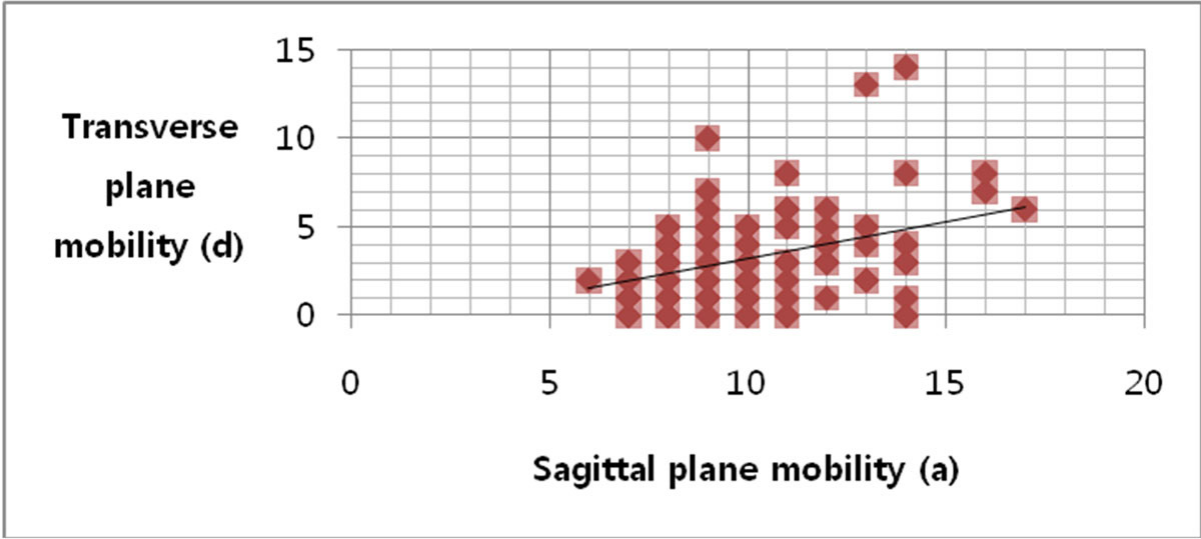


The average of the difference weight bearing and non-weight bearing IMA in the control group was 1.16 and 3.20 in the hallux vallgus group. If we defined 3.6(95 percentile in the normal group) as having axial hypermobility, 42% of hallux valgus patients had first ray axial plan hypermobility (Table 1). The axial plan mobility had no correlation with sagittal plan mobility(Correlation coefficient : 0.25) and also no significant correlation with hallux valgus angle or IMA(Correlation coefficient : 0.5) (Figure 1).

**Table 1:** 

	Difference between weight bearing IMA and non-weight bearing IMA	Sagittal mobility
Hallux valgus group	3.2° (range 0°to 14°)	10.1° (range 7°to 16)

Control group	1.2° (range 0°to 8°)	7.8° (range 6°to 11)

## Conclusion

1st ray axial hypermobility is another disease group in hallux valgus, so should be considered in treatment of hallux valgus.

## Trial registration

Clinical experimental study.
